# Normative Data for Impact Microindentation for Australian Men: Cross‐Sectional Data From the Geelong Osteoporosis Study

**DOI:** 10.1002/jbm4.10384

**Published:** 2020-07-18

**Authors:** Pamela Rufus‐Membere, Kara L Holloway‐Kew, Mark A Kotowicz, Adolfo Diez‐Perez, Julie A Pasco

**Affiliations:** ^1^ School of Medicine, Deakin University Geelong Victoria Australia; ^2^ Department of Medicine‐Western Health Melbourne Medical School, The University of Melbourne Melbourne Victoria Australia; ^3^ Barwon Health Geelong Victoria Australia; ^4^ Department of Internal Medicine Hospital del Mar‐IMIM, Autonomous University of Barcelona and CIBERFES, Instituto Carlos III Madrid Spain

**Keywords:** BONE MATERIAL STRENGTH INDEX, IMPACT MICROINDENTATION, NORMATIVE DATA, OSTEOPOROSIS, POPULATION‐BASED

## Abstract

Impact microindentation (IMI) is a novel technique for assessing the bone material strength index (BMSi) in vivo. However, no studies have presented normative data for BMSi. The aim of this study was to develop such normative data using a population‐based sample of men, randomly selected from electoral rolls for the Barwon Statistical Division in southeastern Australia to participate in the Geelong Osteoporosis Study. BMSi was measured on the tibial plateau using an OsteoProbe in 405 men (ages 33 to 96 years) during the period 2016 to 2019. Associations between BMSi, age, and anthropometry were examined using linear regression models. BMSi values ranged from 49.0 to 100.5. BMSi was negatively correlated with age (*r* = −0.152, *p* = 0.002), weight (*r* = −0.103, *p* = 0.039), and BMI (*r* = −0.187, *p* < 0.001), and positively correlated with height (*r* = +0.107, *p* = 0.032). Mean ± SD BMSi was 82.6 ± 7.0 for the whole group, and ranged from 85.6 ± 6.0 for ages 30 to 39 years to 79.8 ± 6.6 for ages 80+ years. This study provides normative data that can be used to calculate *T‐* and *Z‐*scores for BMSi. These data will be useful for identifying men with low BMSi. Further research is warranted to derive optimal cut points for BMSi that discriminate fracture risk. © 2020 The Authors. *JBMR Plus* published by Wiley Periodicals LLC. on behalf of American Society for Bone and Mineral Research.

## Introduction

Impact microindentation (IMI) uses a novel handheld device, the OsteoProbe, to assess cortical bone in vivo using a minimally invasive method.^(^ ^1^ ^)^ Other devices capable of measuring tissue material properties are mainly intended for application in surgical settings.^(^ ^2^ ^)^ The methodology and unique clinical significance of IMI have been previously reported.^(^ ^3^, ^4^, ^5^ ^)^ During IMI, the more susceptible the bone to fracture, the further the test probe will indent the bone. The technology quantifies bone microindentation distance in relation to a reference material and expresses the ratio as a bone material strength index (BMSi). Hence, lower BMSi values are likely to be associated with an increased risk of fracture. Previous studies have evaluated BMSi in relation to fragility fractures,^(^ ^6^, ^7^, ^8^ ^)^ chronic kidney disease,^(9^ ^)^ and type 2 diabetes.^(^ ^10^, ^11^ ^)^ The clinical nature of such samples and, sometimes, opportunistic samples used as controls, have failed to inform the BMSi values in the underlying populations. It is important to note that the application of inappropriate reference data for clinical assessment of BMSi may bias estimates of fracture risk.^(^ ^12^ ^)^ To our knowledge, no studies to date have reported normative data for BMSi. The aim of this study was to develop age‐related reference ranges for BMSi using an Australian, population‐based sample of men.

## Participants and Methods

### Study participants

Participants for this study were men from the Geelong Osteoporosis Study (GOS), a population‐based cohort study situated in a geographically well‐defined region in southeastern Australia, known as the Barwon Statistical Division.^(^ ^13^ ^)^ The male arm of the GOS commenced in 2001 with recruitment of 1540 men 20 to 92 years old and follow‐up assessments every few years. At least 99% of men recruited for the GOS were white. The inclusion criterion was a listing on the electoral rolls for the region; the only exclusion criterion was inability to provide informed consent to participate in the study. Electoral roll registration is compulsory in Australia; thus, it is a comprehensive sampling frame for adults aged over 18 years. A detailed description of the GOS study design, recruitment, and attrition have been published elsewhere.^(^ ^13^, ^14^ ^)^ Of the 1540 men recruited at baseline, 424 had died before the current follow‐up, 25 had left the region, 8 were unable to provide informed consent, and 188 were unable to be contacted. Of the remaining 895, 233 declined participation citing personal reasons (*n* = 112), time constraints (*n* = 42), old age (*n* = 48), or illness (*n* = 31). Further, at the time of writing, 32 men had not provided clinical measures and 63 men were yet to schedule a follow‐up visit. Thus, data for this cross‐sectional analysis were generated for 567 men (ages 33 to 96 years) assessed in the current follow‐up phase, 2016 to 2019. The study was approved by the Human Research Ethics Committee at Barwon Health (Geelong, Victoria, Australia). All participants provided written informed consent.

### Measures

Height and weight were measured to the nearest 0.1 cm and 0.1 kg, respectively, and BMI (kg/m^2^) calculated. Areal BMD (g/cm^2^) was measured at the femoral neck using DXA (Lunar; Prodigy, Madison, WI, USA). A participant's prior fracture was defined as any low‐trauma fracture equivalent to a fall from a standing height or less, excluding fractures of the toe, skull, finger, and face, occurring during adulthood (age ≥20 years). Fractures were radiologically verified where possible.

IMI was measured using the OsteoProbe RUO (Active Life Technologies, Santa Barbara, CA, USA). The indentation site on the anterior surface of the midtibia was determined by measuring the midpoint from the medial border of the tibial plateau to the distal edge of the medial malleolus.^(^ ^4^ ^)^ Following disinfection of the area and administration of local anesthetic, the OsteoProbe was inserted through the skin and periosteum until reaching the surface of the bone at the anterior face of the midtibia. At least 11 indentations were performed for each participant, of which the first measurement was systematically disregarded to ensure sufficient penetration of the probe through the periosteum, followed by 10 valid test indentations. Two trained operators conducted the IMI measurements following internationally recognized recommendations for using the OsteoProbe^(^ ^4^ ^)^_;_ however, the majority (92.7%) were completed by a single operator. The mean (±SD) BMSi of participants measured by each of the operators was 83.0 ± 7.0 and 82.7 ± 6.9. This was not significantly different (*p* = 0.801). The coefficient of variation (CV) for microindentation was 2% for repeated measures. Precision was calculated as the mean (expressed as %) of SD/mean for two sets of indentations for 10 participants.

We have previously reported the opinions of the men in the GOS regarding their experience with the OsteoProbe, and showed that participants tolerated the procedure well, demonstrating the high feasibility of performing IMI measures.^(^ ^15^ ^)^

### Statistical analysis

BMSi values were normally distributed. Means and SDs were calculated for each age group: 30 to 39, 40 to 49, 50 to 59, 60to 69, 70 to 79, and 80+ years. Relationships between BMSi values and age, weight, height, and BMI were described using Pearson's correlation. Multivariable linear regression models were developed to determine the regression equation and prediction intervals for describing the relationship between BMSi and covariates, and for identifying the best predictors for BMSi. The residuals for the regression models were visualized for normality. Statistical analyses were performed using Minitab V.18 (State College, PA, USA) http://www.minitab.com/en-us/products/minitab/.

## Results

### Participant characteristics

Of 567 potential participants in this current follow‐up, 405 underwent IMI testing. Reasons for nonmeasurement in 162 men were needle phobia (*n* = 22), existing skin infections (*n* = 45), excessive soft tissues around the midtibial region (*n* = 84), discomfort (pressure, not pain) after the first indentation (*n* = 6), inability to provide informed consent (*n* = 2), and three participants did not provide any reasons for declining. Compared with participants, nonparticipants were older (mean ± SD, 70.3 ± 15.9 versus 64.2 ± 11.9 years, *p* < 0.001) and had greater mean BMI (30.2 ± 5.4 versus 27.0 ± 3.2 kg/m^2^, *p* < 0.001).

Participant characteristics are given in Table 1. BMSi values ranged from 49.0 to 100.5. Mean BMSi in the presence or absence of prior fracture was (mean ± SD): 80.0 ± 5.5 versus 82.6 ± 5.9 (*p* = 0.023). No associations were detected between BMSi and BMD (*r* = 0.000, *p* = 0.986).

**Table 1:** Characteristics of All Participants and by Age Groups (*n* = 405)

Age group (years)	*n*	Weight (kg)^a^	Height (cm)^a^	BMI (kg/m^2^)^a^	Femoral neck BMD (g/cm^2^)^a^	Prior fractures (*n*%)^b^
30–39	26	80.4 ± 8.7	178.6 ± 5.5	25.2 ± 2.7	1.031 ± 0.148	1 (0.2)
40–49	56	83.3 ± 14.2	178.3 ± 8.0	26.2 ± 3.7	1.035 ± 0.136	4 (1.0)
50–59	87	83.0 ± 11.2	175.3 ± 6.1	26.9 ± 3.2	1.011 ± 0.138	10 (2.5)
60–69	112	81.8 ± 11.2	173.4 ± 6.1	27.2 ± 3.3	0.963 ± 0.139	11 (2.7)
70–79	81	82.1 ± 10.3	173.2 ± 5.7	27.3 ± 2.9	0.944 ± 0.124	12 (3.0)
80+	43	77.0 ± 9.7	170.2 ± 7.8	26.6 ± 3.0	0.897 ± 0.115	5 (1.2)
All	405	81.7 ± 11.3	174.4 ± 6.9	26.8 ± 3.2	0.971 ± 0.132	43 (10.6)

^a^ Data are shown as mean ± SD.

^b^ Fractures were 3 clinical vertebra, 3 hip, 2 foot, 4 elbow, 5 ankle, 5 humerus, 8 tibia, and 13 rib.

### Associations between BMSi and age

Mean and SD BMSi values for the whole group and according to age‐decades are presented in Table 2. The highest mean BMSi was observed for the youngest age group, 30 to 39 years (85.6 ± 6.0), and the lowest for the oldest age group, 80 years and older (79.8 ± 6.6).

**Table 2:** Bone Material Strength Index (BMSi) by Age Group and for All Participants^a^

Age group (years)	*n*	BMSi
30–39	26	85.6 ± 6.0
40–49	56	82.0 ± 7.6
50–59	87	83.8 ± 6.6
60–69	112	82.9 ± 5.7
70–79	81	82.0 ± 8.3
80+	43	79.8 ± 6.6
All	405	82.6 ± 7.0

^a^ Data are shown as mean ± SD.

**Fig. 1:**
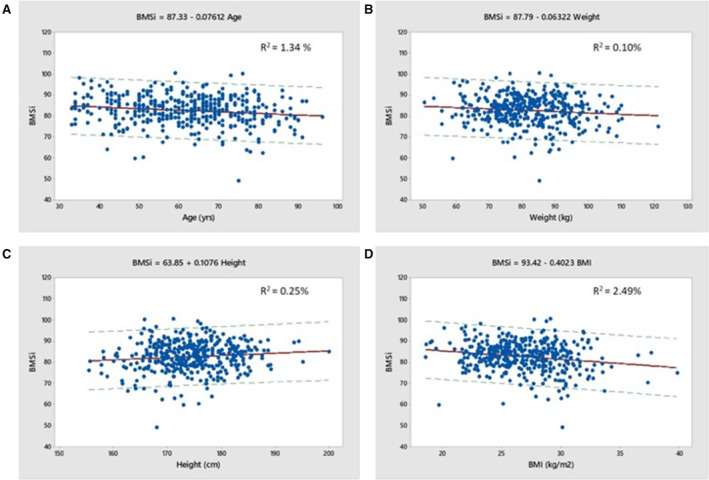
(*A*–*D*) Associations between bone material strength index and age, weight, height, and BMI for men. Regression lines and 95% prediction intervals are shown.

There was a weak negative relationship between BMSi and age across the age range examined (*r* = −0.152, *p* = 0.002) and the slope of the graph indicates that for each decade increase in age, there was a mean 0.8 decrease in BMSi units (Fig. 1 *A*, Table 3).

**Table 3:** Linear Regression Model for Bone Material Strength Index With Age, Weight, Height, and BMI

		β coefficient	SE	*p* Value	*R* ^2^
Model 1	Age (years)	−0.08	0.02	0.002	1.3%
	Constant	87.33	1.56	<0.001	
Model 2	Age (years)	−0.07	0.02	0.008	3.7%
	BMI	−0.37	0.11	0.001	
	Constant	96.52	3.05	<0.001	
Model 3	Age (years)	−0.06	0.03	0.031	3.6%
	Weight (kg)	−0.12	0.03	0.001	
	Height (cm)	0.16	0.06	0.007	
	Constant	67.20	10.10	<0.001	

### Associations between BMSi and anthropometry

BMSi was positively correlated with height (*r* = +0.107, *p* = 0.032) and negatively correlated with weight (*r* = −0.103, *p* = 0.039) and BMI (*r* = −0.187, *p* < 0.001) (Fig. 1 *B*–*D*). In multivariable models, the best predictors of BMSi were age and BMI, which explained 3.7% of the variance (Table 3). BMD did not contribute to the models.

## Discussion

This study presents normative BMSi data for a population‐based sample of men, randomly selected from a geographically well‐defined region in southeastern Australia. BMSi values ranged from 49.0 to 105.0.

In previous clinical studies in patients with specific diseases, BMSi ranges of 60.0 to 87.0 and 70.0 to 89.0 have been reported for women,^(^ ^8^, ^9^, ^16^, ^17^, ^18^ ^)^ and for samples comprised of men and women,^(^ ^6^, ^19^, ^20^, ^21^, ^22^ ^)^ respectively. For control samples in clinical studies, BMSi ranges of 75.0 to 90.0 and 75.0 to 93.0 have been reported for samples involving women^(^ ^8^, ^9^, ^16^, ^17^, ^18^, ^23^ ^)^ and men and women combined,^(^ ^6^, ^19^, ^20^, ^21^, ^22^ ^)^ respectively.

In population‐based studies in women,^(^ ^24^, ^25^, ^26^, ^27^ ^)^ BMSi values ranged from 52.9 to 93.3. However, these studies spanned a relatively narrower age range (75 to 80 years), and there is a dearth of population‐based BMSi data for men. Moreover, in our study, we did not exclude participants on the basis of disease or medication use. The importance of using a large, randomly selected sample has been previously been reported for developing normative data for the broader population.^(^ ^12^ ^)^

We report a small, age‐related decline in BMSi for each age‐decade increase: BMSi declined by approximately 0.8 units. Such a decline seems reasonable, given that the main determinants of bone strength (bone mass and bone structure) also deteriorate with increasing age.^(^ ^28^, ^29^ ^)^ The differences in bone material properties observed with increasing age in this study might be explained by the age‐related accumulation of microcracks and a deterioration in bone microarchitecture.^(^ ^30^, ^31^ ^)^ Consistent with our data, Malgo and colleagues^(^ ^21^ ^)^ reported an inverse association between age and BMSi for 90 patients (male and female) with low bone mass (*r* = −0.539, *p* < 0.001). In contrast, Duarte Sosa and colleagues^(^ ^23^ ^)^ and Popp and colleagues^(^ ^17^ ^)^ reported no association between age and BMSi in their case–control studies of postmenopausal women investigating fractures and bisphosphonate treatment. Moreover, we have previously reported no correlation between age and BMSi in a subset of this cohort; this is likely to be a type 2 error as the number of participants was smaller than in this analysis.^(^ ^15^ ^)^ The relatively smaller number of men in the 40 to 49 years category may also explain why the same mean BMSi values were observed in men aged 40 to 49 years and those aged 70 to 79 years.

Thus, the lack of consistency in the literature may be partly explained by the number of participants, the range of ages, and sampling frames of the participants studied.

BMSi was negatively correlated with weight and BMI, and positively correlated with height. Sundh and colleagues^(^ ^25^ ^)^ also reported a negative correlation *(r* = −0.17, *p* = 0.01) between BMSi and BMI in a population‐based study of 202 women between 75 and 80 years of age; Rudang and colleagues^(^ ^24^ ^)^ reported a weak negative correlation *(r* = −0.14, *p* = 0.04) between BMSi and weight in a sample of 211 women between 75 and 80 years of age, and no association with height. Popp and colleagues^(^ ^17^ ^)^ observed no association between BMSi and BMI (*r* = −0.08, *p* = 0.31) in a sample of 153 postmenopausal women. To the best of our knowledge, no studies have explored the relationship between BMSi, weight, and BMI in men only.

Reports on the effects of high BMI and adiposity on fracture risk are equivocal. Some studies suggest that a high BMI may be associated with increased fracture risk.^(^ ^32^, ^33^, ^34^ ^)^ After BMD adjustment, obesity appears to contribute to increased fracture risk,^(^ ^35^ ^)^ suggesting that other mechanisms such as poor bone quality or increased falls risk may be responsible for the deleterious effects of excessive adipose tissue.^(^ ^25^ ^)^ Moreover, a site‐dependent association between obesity and fracture has been reported in women, with higher fractures occurring at sites with more cortical bone, including the ankle and upper arm.^(^ ^25^, ^33^, ^36^ ^)^ Conversely, Premaor and colleagues^(^ ^34^ ^)^ have reported a decreased fracture risk in most sites in men with obesity. Few studies have investigated the relationship between BMSi and height. At this stage, we have no clear explanation about the correlation between BMSi and height based on our results.

However, various mechanisms through which height may influence bone properties include hip axis and moment arm length,^(37^ ^)^ as well as bone structure, particularly in long bones.^(^ ^38^ ^)^ Thus, further studies are warranted to investigate the relationship between BMSi and different aspects of anthropometry.

The major strength of this study is that data were obtained from a randomly selected sample from the population of southeastern Australia; thus, it will be relevant for the underlying population. We have also provided mean and SD values for each decade, which can be used to calculate *T‐* and *Z*‐scores for BMSi. Furthermore, we explored the associations between BMSi, age, and anthropometry in the largest sample, and widest age range of men to date. Notwithstanding, loss to follow‐up and exclusions for BMSi testing limited our ability to explore associations across the full range of BMI, as excessive accumulation of soft tissue at the measurement site was the most common reason for nonparticipation.

In the only published study evaluating geographical variation in BMSi, significant differences in BMSi were observed between countries, with BMSi higher in healthy Spanish women than in healthy Norwegian women.^(^ ^23^ ^)^ This indicates that the observations from this study may not be generalizable to other populations, as there may be differences in BMSi values between geographical areas. Comparable data will be needed for other geographical regions. Moreover, although the assessment of only male subjects may limit the transferability of these findings, prior studies^(^ ^6^, ^7^, ^20^, ^21^, ^39^ ^)^ suggest that sex is not associated with varying BMSi values.

To the best of our knowledge, this study provides the first normative data for BMSi in men; these data will be useful for identifying BMSi deficits in men. Further research is warranted to derive optimal cut points for BMSi that discriminate fracture risk in this population.

## Disclosures

PGR is supported by Deakin University Postgraduate Industry Research Scholarship. KLH‐K is supported by an Alfred Deakin Postdoctoral Research Fellowship. AD‐P owns shares of Active Life Scientific, Inc., the manufacturer of the RPI device. MAK and JAP are recipients of grants from the NHMRC; KLH‐K, MAK, and JAP are recipients of a grant from Amgen‐GSK OA‐ANZBMS and Amgen Australia.

## Peer Review

The peer review history for this article is available at https://publons.com/publon/10.1002/jbm4.10384.
